# Investigating the national implementation of SMS and mobile messaging in population screening (The SIPS study)

**DOI:** 10.1016/j.ebiom.2023.104685

**Published:** 2023-06-27

**Authors:** Amish Acharya, Gaby Judah, Hutan Ashrafian, Viknesh Sounderajah, Nick Johnstone-Waddell, Mike Harris, Anne Stevenson, Ara Darzi

**Affiliations:** a Institute of Global Health Innovation, Imperial College London, London, W2 1NY, United Kingdom; b Department of Health and Social Care, London, SW1H 0EU, United Kingdom; c Public Health England, London, United Kingdom

**Keywords:** Mobile messaging, Digital communication, Population screening, SMS, Implementation

## Abstract

**Background:**

The increasing use of mobile messaging within healthcare, poses challenges for screening programmes, which involve communicating with large, diverse populations. This modified Delphi study aimed to create guidance regarding the use of mobile messaging for screening programmes, to facilitate greater, and equitable screening uptake.

**Methods:**

Initial recommendations were derived from a literature review, expert scoping questionnaire, public consultation, and discussion with relevant national organisations. Experts from the fields of public health, screening commissioning, industry and academia voted upon the importance and feasibility of these recommendations across two consensus rounds, using a 5-point Likert scale. Items reaching consensus, defined *a priori* at 70%, on importance and feasibility formed ‘core’ recommendations. Those reaching this threshold on importance only, were labelled ‘desirable’. All items were subsequently discussed at an expert meeting to confirm suitability.

**Findings:**

Of the initial 101 items, 23 reached consensus regarding importance and feasibility. These ‘core’ items were divided across six domains: message content, timing, delivery, evaluation, security, and research considerations. ‘Core’ items such as explicitly specifying the sender and the role of patient involvement in development of screening message research had the highest agreement. A further 17 ‘desirable’ items reached consensus regarding importance, but not feasibility, including the integration into GP services to enable telephone verification.

**Interpretation:**

These findings forming national guidance for services, will enable programmes to overcome implementation challenges and facilitate uptake of screening invitations. By providing a list of desirable items, this study provides areas for future consideration, as technological innovation in messaging continues to grow.

**Funding:**

10.13039/501100013631NIHR Imperial Patient Safety Translational Research Centre.

**Research in context**

**Evidence before this study**

A search of PubMed, Medline, EMBASE and Google scholar databases for literature published from 1st January 2000 was undertaken. Terms including *mass screening, population screening, abdominal aortic aneurysm, diabetic retinopathy, cervical cancer, cervical smear, mammography, colonoscopy, sigmoidoscopy, newborn hearing*, *thalassemia, sickle cell, congenital abnormalities, lung cancer* in conjunction with *mobile, cell, phone messaging, Short Message Service, Multimedia Message Service* and *text messages.* Additional articles relevant to healthcare messaging were also found using grey literature, including publications from Public Health England and the US Food and Drug Administration (FDA). All articles were screened by two independent authors, with disagreements discussed. 44 articles were included, of which 6 were guidance or practice points. Most studies reported interventions involving changing the content of messaging to improve attendance. This included the use of SMS messaging as appointment reminders and in health promotion activities. Security concerns were also reported within practice guidelines, including techniques to avoid confidentiality breaches. These predominantly focussed upon the use of messaging within isolated contexts such as primary care. To a lesser extent the literature also highlighted message timing, delivery and evaluation as considerations, however, none examined mobile messaging at a population-level. There was therefore a paucity of robust guidance for such programmes, and none specifically examined how future advances will impact health communication.

**Added value of this study**

We provide an evidence-derived guidance on how population screening programmes can effectively utilise mobile messaging. In doing so, we address key gaps in the literature, such as identifying robust evaluation metrics, means of delivery and effective messaging schedules to facilitate an effective implementation of these communication tools. This study overcomes the limitations within the existing literature which focusses upon singular healthcare contexts, or isolated patient groups. Moreover, unlike the existing literature, we identify ‘desirable’ items, which although currently not feasible, sets the agenda for future research, including leveraging the advancements within messaging technology.

**Implications of all the available evidence**

Our findings suggest that there are core components of mobile messaging that need to be considered for its effective implementation into screening programmes. Moreover, in conjunction with the existing literature, the study suggests utilising non-coercive simple language involving behavioural science informed content may facilitate uptake. Whilst, incorporating robust impact assessments examining healthcare inequalities, can ensure messaging can facilitate equitable screening practices. More work is needed, however, to explore whether the needs of individual screening programmes are met following these findings.

## Introduction

Mobile messaging encompasses a range of text and multimedia platforms delivered through mobile devices including Short Message Service (SMS) and Rich Communication Services (RCS).^1^ Within healthcare the use of these technologies has become ubiquitous, with approximately 40% of UK General Practitioners (GP) using text messaging regularly in 2017, to communicate with patients.^2^ This popularity stems from the fact that mobile messaging is viewed as both acceptable and reliable by patients. It is also an extremely versatile means of communication, having been implemented successfully in a diverse range of clinical fields including pain management and dietetics.^3^^,^^4^ The use of health mobile messaging is likely to grow further following the significant role it has played in the coordination of public health responses during the COVID-19 pandemic, and as smartphone penetrance increases.^5^^,^^6^

Population screening programmes, unlike other areas, however, pose unique challenges to healthcare messaging, and mobile services. In the UK, there are currently 11 active population screening programmes, inviting approximately 15 million people annually.^7^ These large populations have differing communication needs and levels of awareness of screening services. If screening programmes do not account for the varying health and digital literacy of these groups when sending mobile messaging, they may exacerbate existing inequalities. In addition, several programmes, for example breast cancer screening, are administered through regional hubs, as opposed to known healthcare services such as GP practices.^8^ An individual may therefore be contacted by a service they have never interacted with previously and may lack trust in the provenance and content of the message. Increased information security concerns arising during the COVID-19 pandemic are likely to have compounded this mistrust. The Information Commissioner's Office (ICO), reported 74% more health-related cyber security incidents between April and June 2021, compared to the same period in 2020.^9^ Finally, evaluating the impact of screening messaging also requires more consideration. Unlike other healthcare scenarios where a patient will have requested an appointment directly or by-proxy (e.g. through referral), screening invitations are sent to an asymptomatic population at pre-defined intervals. Measuring the effect of message content solely through attendance, may ignore an individual's informed choice not to attend, with a survey showing 90% of the population agree with cancer screening.^10^ More holistic measures of effectiveness are therefore needed in these contexts.

Of the few available frameworks regarding the implementation of mobile messaging in healthcare, the predominant focus of guidance has been upon a single service (e.g. a GP practice) or examines optimal content only.^11^, ^12^, ^13^ For example, guidance by the NHS Transformation Directorate for healthcare workers, focussed upon where to present transparency information, general confidentiality concerns and recording information.^12^ It did not, however, examine how to ensure telephone numbers are appropriately verified before sending messages, which is important for screening programmes which utilise their own databases for service users, or are reliant on external health records being updated. Other guidance documents such as CRISP, do provide a toolkit on the platform considerations and messaging components such as short and long codes,^14^ however, these are more relevant to a single service e.g. GP practice implementing mobile messaging. The guidance does not give information on optimal timing of messages, which is important given the reliance of several screening programmes on reminders. Furthermore, it does not give recommendations on the use of content such as GP endorsement, which is a particularly important measure that can potentially help to improve the uptake of screening appointments.^15^ Although existing frameworks provide some insight which may be applicable, no document addresses the breadth of considerations, including security and delivery of messages, nor depth of content information, such as how to make messaging accessible to diverse groups, required by population screening programmes. The aim of this study, therefore, is to utilise a modified Delphi approach to understand the breadth of issues regarding the use of mobile messaging by screening programmes, and the key considerations regarding implementation. Through this process, the study will develop a list of expert-derived core recommendations for services, as well as highlight areas to inform the future directions of mobile messaging in this context.

## Methods

**Fig. 1:**
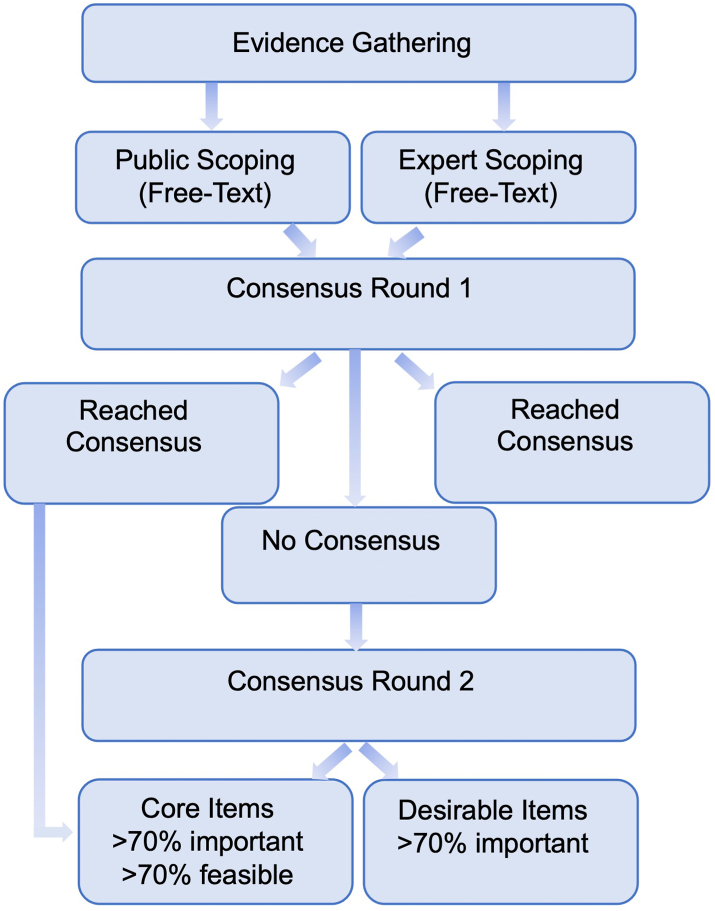
Study diagram demonstrating the phases of the study including evidence synthesis and consensus rounds.

The study was conducted in two distinct phases: evidence synthesis and consensus (Fig. 1). The steering committee was convened by the principal investigator (AD) to ensure representation from collaborating organisations. The full methodology and rationale are provided in the published protocol for this study and are available via its online registration https://osf.io/stf49/.^16^^,^^17^

### Evidence synthesis

During the evidence synthesis a literature review of academic and grey literature was undertaken to elicit the key considerations regarding implementation, and any areas of contention regarding the use of mobile messaging in screening programmes. App-based messaging was included within the search strategy as there was overlap in several areas such as message content, timing, and evaluation. These could provide insights for screening services, however the primary aim of this study was not to provide App-based messaging guidance, which is currently not being used by programmes. Two independent authors were responsible for extracting data, which was used by the steering group to devise recommendations or items within an initial scoping questionnaire. Prompts, such as ‘ideal message length’ were provided within the online questionnaire to elicit free-text responses from experts and members of the public regarding a broad range of topics. The questionnaire has been provided in the study protocol.^14^ Experts were individuals the steering group determined had significant experience in the intersecting fields of UK-based screening programmes, public health, health communication, industry, and academia. Free-text responses from both experts and the public were analysed using an inductive thematic approach, using NVivo qualitative data analysis software (QSR International Pty Ltd, 2021) in which two authors familiarised themselves with responses, then subsequently sorted and coded them. Codes were then discussed, with disagreements resolved, with recurring themes and sub-themes elicited. Sub-themes were further discussed amongst the wider steering group and an item list generated. Items were worded as recommendations, with one or more items relating to each elicited sub-theme. The steering committee discussed the results of the thematic analysis to formulate draft wording for each recommendations. The original information sources used within the extraction exercise were also referred to when drafting this wording. These are demonstrated in the supplementary material.^11^, ^12^, ^13^, ^14^, ^15^^,^^18^, ^19^, ^20^, ^21^, ^22^, ^23^, ^24^, ^25^ During this drafting exercise the steering committee examined the amounts of evidence pertaining to a particular item (e.g. code frequency, number of information sources stating the recommendations), the presence of contradictory information from either the scoping or existing evidence and the quality of the evidence underpinning the item (randomised trial, respected healthcare authority). Items which had contradictory, or consistent low-quality evidence behind them were not drafted into recommendations. Items which did not currently have substantial evidence underpinning their inclusion, but were considered important considerations from the scoping exercise, particularly if they pertained to the potential future developments in screening messaging were also drafted into recommendations. Wording was selected by means of a consensus approach, and was agreed upon by the steering committee to reflect the varies pieces of evidence from which they were derived.

### Patient and public involvement and engagement (PPIE)

Due to the public-facing nature of mobile messages from screening programmes, significant PPIE input was gathered. Members of the public were recruited through the Patient Experience Research Centre (PERC) at Imperial College London, which is a department specialising in helping members of the public engage with research, and who work closely with local community organisations. In addition, recruitment was undertaken using the online platform VOICE (Newcastle, UK), which is a large online community for public engagement involving the general public, as well as patients and those with lived experience of health conditions. Although purposive sampling techniques were not utilised, PPIE organisations had a diverse membership from which to draw from to ensure a range of experiences was included. A target sample size of 20 was chosen in keeping with similar Delphi studies.^26^ No restriction was placed upon the demographics of the public group or experts, and all demographic measures including gender were self-reported. The public group were asked to provide opinions on what aspects of messaging they felt were important. They were also asked to provide feedback on the preliminary item list and make suggestions regarding additional items to incorporate. Responses from the public groups were analysed both qualitatively (through an inductive thematic analysis as described previously) and quantitatively (aggregation of responses e.g. 60% of the public group highlighted simple language as an important consideration). Findings were made available to experts in subsequent consensus rounds.

### Consensus rounds

Two online consensus rounds were conducted to determine which items should be considered for inclusion in the final recommendations using the Qualtrics XM platform (Qualtrics, Provo, UT). During each of these rounds, experts were asked to vote, using a 5-point Likert scale (e.g. 1-*extremely unimportant and* 5-*extremely important)*, whether an item was important to consider when using mobile messaging in screening. They were also asked to vote upon the current feasibility of implementing each recommendation, again using a 5-point Likert scale (e.g. 1-*absolutely unfeasible and* 5-*absolutely feasible)*. The nature of feasibility was not defined, but was suggested to include characteristics such as cost-effectiveness, technological capability, and sustainability. Consensus was defined *a priori* at 70%, in keeping with other comparable Delphi studies.^27^ To aid consensus, feedback was provided to experts from previous voting rounds (for the second consensus round), the evidence synthesis, feedback from the public groups and from specialist organisations such as the National Cyber Security Centre (NCSC). The NCSC were consulted to examine the item list to ensure compliance with other recommendations surrounding data security.

Items which reached the consensus threshold of 70% with respect importance and feasibility after either voting round were designated as ‘core’ items. Those reaching consensus with respect importance but not feasibility were labelled as ‘desirable’ for the final meeting. Items where less than 70% of the experts agreed they were important, were voted upon in a second round of voting, as were any new items suggested during the first round. The second consensus round followed the same format as the first, with items that still did not reach consensus discussed at the final consensus meeting. The aim of this meeting, held online due to the constraints of COVID-19, was to develop finalised core guidance, review desirable recommendations and discuss items which had not reached consensus. During this meeting the wording of recommendations was also revisited by participants. Suggestions were made to amend the wording to more accurately reflect practice within screening services, with changes made if a majority agreed. A more granular measure of ‘strength’ of each recommendation, beyond ‘core’ and ‘desirable’ items was not provided as it was felt to be beyond the scope of these practicable considerations for active screening programmes. Moreover, not all items were relevant to each screening programme, nor had all items been investigated within each programme. It was therefore considered inappropriate to draw overarching conclusions regarding the overall strength of each recommendation, however rates of agreement are provided in the supplementary material.

### Statistics

As this was a modified Delphi study, no statistical analyses were undertaken. Voting frequency was aggregated and converted to percentages regarding importance and feasibility.

### Ethics

The Institutional Review Board at Imperial College London granted ethical approval for this study (reference 20IC6088). All participants (public and experts) gave informed consent for each part of the study and had the freedom to withdraw at any stage.

### Role of funders

The research was supported by the 10.13039/501100000272National Institute for Health Research (NIHR) 10.13039/100013216Imperial 10.13039/501100013631Patient Safety Translational Research Centre. The funder had no role in the conception, undertaking or publication of this work. The views expressed are those of the authors and not necessarily those of the NHS, the NIHR or the Department of Health and Social Care.

## Results

**Fig. 2:**
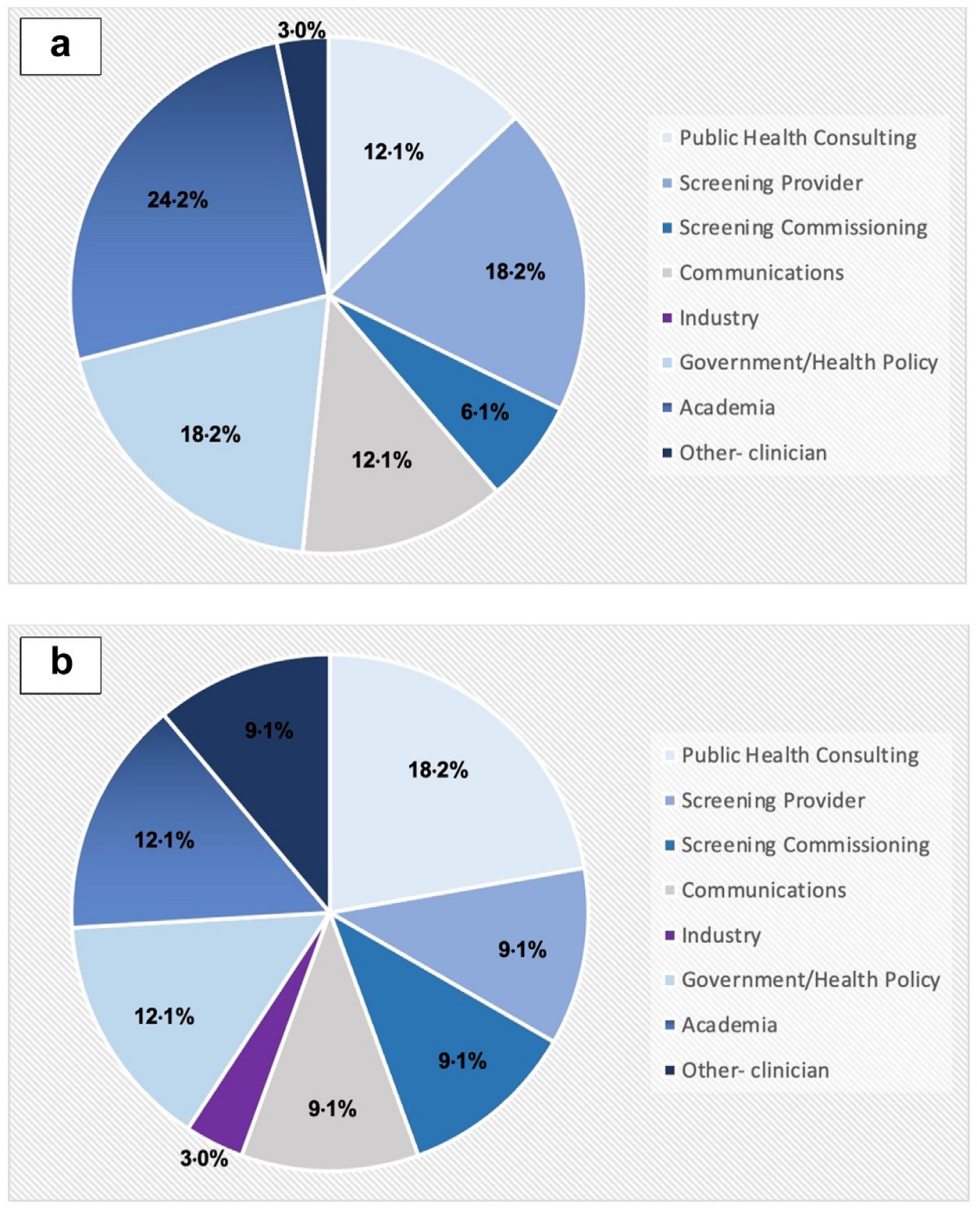
(a) Primary area of experience of members of the expert panel. (b) Secondary area of experience of members of the expert panel.

A total of 33 experts who between them had experience of all 11 active adult population screening programmes in the UK were recruited out of 50 who were approached. No differences were noted in the programmes that they represented. Further demographic comparison of those who did, and those who did not participate was precluded, as those who did not consent to participate did not complete questionnaires. Furthermore, 6.1% of experts had experience with the lung health check, a currently piloted screening programme which aims to detect lung cancer amongst current and ex-smokers. The primary and secondary areas of expertise of this group are shown in Fig. 2a and b respectively. The majority of experts had primary expertise within academia (24.2%), with a further 18.2% in UK screening provision, 18.2% in government health policy and 12.1% in communications and 12.1% in public health**.** All experts had experience using mobile messaging, 69.7% (23 out of 33) had experience using it for personal communication, 51.5% (17 out of 33) for health communication, 42.4% (14 out of 33) for professional communication and 24.2% (8 out of 33) had conducted research into mobile health messaging.

In addition, 22 members of the public participated in the initial scoping. All public participants had either been invited to, or were familiar with, one of more of the UK screening programmes. The age of public participants ranged between 18 and 71 (median 51), with the majority female (63.6%) and all were resident in England at the time of the study. No public participants were familiar with the new-born blood spot or the currently piloted lung health check. Whilst most public members had received reminders for health appointments (72.7%) or had experience using mobile messaging for personal communication (68.2%), only 4.5% had used it to send information to healthcare professionals.

Following the evidence synthesis, and inductive thematic analysis of free-text responses, six broad domains of interest were highlighted: message content, timing, delivery, evaluation, security/governance, and research/future considerations. Items were mapped into the six broad themes elicited from the thematic analysis during the consensus rounds. One hundred and one items were voted upon by experts during the first consensus round, of which twenty-eight achieved consensus to be included without further voting (importance >70%). A further fifty-four did not reach consensus and were voted upon in the second round. Twelve items were included from this round, with the remaining forty-two still not reaching consensus. Across each voting round 100% of experts responded. Cronbach's alpha was 0.966 and 0.778 following the first and second consensus rounds respectively, indicating high inter-rater reliability. The consensus meeting was attended by 29 experts online. There were no changes to which items were included into the final list, however the wording of some were amended to better reflect current practice. Moreover, it was decided to have experts vote upon which future technologies (e.g. push notifications, app integration) were considered the highest priority for services to investigate further. These items had originally been listed individually within the initial voting list but had not reached consensus. A list of all items that were voted upon has been provided in the supplementary material. A full list of items which reached consensus can be found in Table 1.

**Table 1:** Demonstrating the final items recommended by the expert panel regarding the use of mobile messaging in population screening programmes.

**Content**	
1. Using concise simple language (reading age of 9)	☑
2. Using non-technical language with factual, non-coercive information	☑
3. Specifying the date, time (am/pm), location	☑
4. Include additional information such as what to bring, or what to do, where possible.	☑
5. Specifying who has sent the message (e.g. screening service or GP practice) and purpose	☑
6. Including weblinks to evidence or more information (e.g. screening website)	☑
7. Providing a telephone number to book	☑
8. Where appropriate using GP endorsement in reminder messages (e.g. [Practice name] encourages you to screen]	☑
9. Sending messages to facilitate attendance at screening (without being coercive), which could use behavioural science	☑
10. Using Did Not Attend Messaging (DNA) messages for missed appointments	☑
11. Sending messages in English, but with language translations available (e.g. via weblink or by previous selection)	★
12. Providing an ability to re-book in the message other than telephone no. (e.g. by text or weblink)	★
13. Using messages tailored or targeted at certain groups (such as patients at higher risk of an illness)	★
**Timing**	
1. 2 messages maximum should be sent at 1 time in the programme ideally	☑
2. BEFORE an appointment 2 reminder messages should be sent at day 7 before then at day 2 before.	☑
3. FOLLOWING an open invitation (e.g. to book an appointment) or sending of testing kit (e.g. FIT) 3 messages should be sent if there has been no booking or returned kit. These will be on average 12 days, 20 days then 28 days after the invitation.	☑
4. Using confirmation texts immediately if a booking has been made or a kit has been received	★
**Delivery**	
1. Flagging individuals who have who it might not be appropriate to message (e.g. following a miscarriage/patient passing away)	★
2. Ensuring all services are integrated into the GP Spine to enable telephone number verification	★
3. Verifying numbers through direct contact with patients where possible	★
4. Enabling limited bi-directional messaging service (e.g. for functions such as booking, confirming locations, organizing translated messages)	★
**Evaluation**	
1. Routinely evaluating the impact of new/different messages on regional healthcare inequalities	☑
2. Measuring user satisfaction by recording opt-out rates	☑
3. If no existing pathway is available, periodically assessing usefulness of messages/satisfaction through other means (online, telephone and in writing)	☑
4. To ensure ongoing acceptability of messages to the public, introducing ongoing testing (e.g. online A/B testing, or User-experience trials)	☑
5. Incorporating satisfaction measures into existing pathways (e.g. GP practices or NHSP Parent Survey) where possible	★
6. Assessing measure mobile message delivery success reports and measure responses rates (e.g. in bi-directional messages, or appointment calls)	★
7. When necessary using linked datasets (e.g. between screening services and GP data or hospital data) to facilitate the evaluation on healthcare inequalities	★
8. Routinely collect measures of knowledge and attitudes (e.g. Decisional Conflict Scale) to screening to determine the effect on informed choice	★
**Security**	
1. Maintaining consistency across media including publishing contact details/links on websites and in letters, so individuals can verify these as legitimate	☑
2. Using MEF-registered (official) SenderIDs (e.g. “[Screeningservice] sent you a message”, as opposed to “[+4478 …] sent you a message”)	★
3. Defining a wrong recipient message receipt as a reportable breach	★
**Research & future**	
1. Using experimental methods such as Randomised Controlled Trials to determine the impact of novel messages	☑
2. Using online experimental methods such as A/B testing to determine the impact of novel message	☑
3. Routinely report the outcomes of trials/research on population inequalities (e.g. between different demographics, and individuals with different health conditions)	☑
4. Prior to large trials, new messages should ensure Patient and Public Involvement and qualitative measures are undertaken	☑
5. Screening services/PHE Publishing their research priorities, to enable researchers to focus upon relevant areas (this includes non-content related areas)	☑
6. Involving top-down infrastructure and governance support to facilitate research, including enabling trials across services/regions e.g. providing roadmaps for trial conduct, dissemination findings to stakeholders	★
7. Implementing fast–track processes to enable widespread testing for messages with trial evidence	★
8. Facilitate the examination of new technologies e.g NHS approved app-based integration or push notifications	★

☑ Core item (reached consensus as important and feasible).

★ Desirable item (reached consensus as important, but not feasible).

### Content

The majority of existing work into the use of messaging in screening has focussed upon content. Experts agreed that making message content accessible to those with diverse communication needs was important and could feasibly be undertaken. 87.9% felt using language suitable for individuals with a reading age of 9 was important whilst 83.9% felt it could feasibly be incorporated (Table 1). Experts also agreed that the use of behavioural science-informed messages, and particularly GP endorsement, was an important and feasible means of facilitating screening attendance. These messages have predominantly been utilised in research settings; however, this consensus provides a stronger mandate to translate findings into practice. In the future this content could be tailored or targeted based upon risk or socio-demographic factors, which experts felt was desirable, but currently unachievable due to data quality preventing accurate population segmentation. For example, experts felt that some factors such as ethnicity were poorly coded in current healthcare records to prevent targeting. In other cases although data was available as the screening service is administered separately to primary and secondary care e.g. breast screening, services did not have access to the necessary data to segment messaging.

### Timing

Messages are often used by screening services as reminders. 78.8% of experts agreed it was important to limit reminders to a maximum length of 320 characters (or equivalent to two messages). Panellists agreed that sending a maximal two reminder messages seven days and again two days prior to a pre-booked appointment (e.g. a screening mammogram) was optimal. It was also determined that sending three reminders at 12 days, 20 days and 28 days after a written invitation was best practice if an open invitation had not been responded to, or self-sampling kit had not been returned (e.g. with FIT testing in bowel screening).

### Delivery

The delivery methods of mobile messaging in screening programmes is currently limited, with SMS messaging the predominant modality. With growing functionality from Rich Communication Services (RCS), and app-based messaging, experts felt whilst introducing bi-directional messaging would be beneficial it was not yet feasible. In addition, the group felt the ability for services to flag individuals whom it would be inappropriate to contact (e.g. those whom are deceased), and improving telephone number verification through cross-service integration (e.g. with the GP Spine) would be desirable. Although users may ignore messages received whilst abroad, experts did not feel that using International Mobile Subscriber Identifier (IMSI) to determine the roaming status of recipients was necessary.

### Evaluation

The panel acknowledged the importance of a holistic evaluation of messages beyond standard metrics such as read receipts. Where current means to determine user satisfaction have not been implemented, panellists agreed programmes should periodically assess this through other means, with support for A/B testing and user-experience trials. In addition, there was a consensus that evaluation should focus upon how mobile messages impact healthcare disparities and informed choice. Experts agreed the use of linked population datasets could facilitate this analysis but agreed that data access was limited making this currently unfeasible, but important.

### Security/governance

Security and mistrust of mobile messaging can limit its role in health messaging. This of increasing concern in the wake of public health messaging sent during the COVID-19 pandemic, in which there were reports of phishing attacks exploiting health messaging. Experts agreed that maintaining consistency across media, for example invitation letters, website and information leaflets was key at enabling individuals to verify the veracity of information sent by mobile messages. Furthermore, using Mobile Ecosystem Forum (MEF) registered identifiers (IDs) although considered important to prevent fraudulent activity such as phishing, could not be ubiquitously introduced, so were considered currently unfeasible. To maintain trust experts also considered it important to report wrong recipient instances as data breaches, but was only considered a desirable item as it would require a robust national infrastructure.

### Research and future considerations

In keeping with existing national priorities, 78.9% of experts agreed research trials involving mobile messaging and population screening, should report the effects upon healthcare inequalities and population sub-groups. There was also consensus regarding the importance of including and reporting PPIE work in such research. There was, however, a discrepancy regarding the extent to which national bodies and services would collaborate with research. Whilst 84.8% of experts agreed it was important, and 79.1% agreed it was feasible for screening services to routinely publish screening priorities to guide research, having national organisations provide the infrastructure to facilitate research processes did not reach consensus with feasibility. For example, in terms of assisting the dissemination of research findings and supporting the widespread testing of mobile messaging with trial evidence, only 60.6% believed this would be feasible. Following the initial item list and feedback from experts, during the consensus meeting panellists were asked to vote for potential future technological considerations that may improve screening mobile messaging. The use of app-based messaging was the most commonly chosen future priority with 27.6% votes. The use of push notifications and integration into NHS app were also commonly selected, each with 20.7% of the votes (Table 2).

**Table 2:** Mobile technologies that experts felt should be investigated for future implementation in screening.

Future Technological Consideration	Experts voting this as an important priority (%)
App-based messaging	27.6
Push notifications	20.7
Integration into personal calendars/NHS app	20.7
Multimedia messages	17.2
Bot technology	6.9
Other: “ability to select preferences”	3.4

## Discussion

Population screening poses unique challenges for the use of mobile messaging. This study provides robust recommendations to facilitate the implementation of mobile messaging in population screening programmes. By using a modified Delphi method, we highlight 23 core recommendations on the use of mobile messaging, which experts consider to be both important and feasible for screening programmes. These core principles, across six domains (message content, timing, delivery, evaluation, security/governance, and research/future considerations), provide a practicable means of implementation and enables services to gain the most benefit from mobile messaging services. Our findings show that services will not only need to consider content, which is the often the predominant focus, but also on the timing, delivery, evaluation, security, and research of messages. Furthermore, this study has provided 17 desirable principles, which although important, may not be feasible within the current screening infrastructure. These desirable items provide future directions for the use of mobile messaging by services and include a list of technological developments that should be the focus for future implementation.

Mobile messaging has become an increasingly utilised tool in healthcare, in keeping with the increase in smart phone ownership, estimated to have grown by 5 times between 2008 and 2021.^5^ Given this rise in usage, effective communication is essential, especially in screening programmes who are provide services to large, diverse populations. The current principles advocate this use of plain-language, increasing information availability, and incorporating behavioural science-constructs including increasing perceived benefits of screening, which studies have shown improve the receptiveness and understanding of message recipients.^28^ This can improve service-user experience and facilitate attendance. For example, several screening programmes such as bowel, breast and aneurysm screening focus upon older cohorts, however, mobile phone usage is lower amongst these groups. Studies have shown that older adults often face barriers to attendance with respect awareness of continuing to screen, negative experiences of screening and a need for more tailored content.^29^ As shown by the current study, mobile messaging provides the versatility to address these needs. Core recommendations such as the inclusion of non-coercive behavioural science techniques may help to reduce negative emotions towards attendance, whilst the provision of links to additional materials, can enable participants to be better educated about screening.^30^^,^^31^ Moreover, the incorporation of more desirable items such as the integration of targeted message services may also enable risk and age-stratified information to be delivered directly to older adults, which not only can improve knowledge, but also facilitate attendance.^32^

The validity of our findings is supported through our recruitment of a large group of experts from the intersecting fields of screening, public health, communications, and academia. However, our findings must be taken into consideration with the limitations of study. Although the modified Delphi technique has been utilised extensively in healthcare research, it has been subject to criticism regarding the reproducibility of results and attrition biases.^33^^,^^34^ To mitigate the former, the expert panel had significant cross-programme experience of all 11 adult screening services. To ensure we represented a diverse array of perspectives recruitment of experts required agreement between the steering group that individuals had significant experience in their field. A purposive sampling technique was used to ensure there was representation across programmes within different disciplines, although this was skewed toward cancer screening more than other programmes. Furthermore, we involved a PPIE group to ensure our findings incorporate public opinion of mobile messaging. Moreover, through our consensus rounds, there was no loss of participants, demonstrating the high engagement we had with this subject matter; however, our study is still reliant upon the perspectives of only a subset of stakeholders involved with national screening.

Potential weaknesses, for example the inclusion of a subset of experts, may impact upon the generalisability of our findings. Although experts were selected due their breadth of experience in public health, screening, and communications, it cannot be assumed that they were aware of all the aspects discussed. This includes items such as timing, which there is currently a paucity of evidence underpinning, and experts may therefore be unaware of best practice. Experts' opinions may therefore be biased regarding their own impressions of the best timing. However, such limitations were mitigated by undertaking a robust evidence generation exercise, and where possible involving expert partner organisations. This evidence synthesis included not only an evaluation of current standards, but engaged with stakeholders and service users, to gain additional relevant insights. However, a further limitation that must be acknowledged is only a small public group was included. Whilst this is similar to existing Delphi studies, and the involvement of patient organisation ensured a broad reach, this cannot be assumed to be completely representative of the public perceptions. Future work will look to validate this recommendation in a wider public feedback exercise, involving the development of potential template messages. Some of the core principles we have elicited, such as the need for jargon-free text or the verification of recipient contact details, are generalisable to all types of healthcare communication, and have been highlighted in related guidance for practitioners.^14^^,^^23^ However, we have also presented screening-specific recommendations, not currently reported in existing guidance, which may help navigate the complexity of communication for these programmes. These recommendations are underpinned by the best available evidence and leverage the experience of exerts working in screening and allied fields to ensure they are practicable. These specific items include the need to identify who is sending the message because patients may not have knowledge of a screening programme. This is particularly important as studies have shown that knowledge of services such as breast and cervical cancer is 15% lower amongst minority ethnic groups.^35^ Furthermore, this potential lack of knowledge highlights the importance of including measures to improve public trust that screening messages are authentic. Such measures include the recommendation that services maintain consistency (e.g. in language/branding/content provided) across all media including messages, websites and written communication, which has been shown to increase people's responsiveness to public health messaging,^36^ and the suggestion to use MEF-registered sender IDs which is a cross-industry platform to avoid phishing.^37^ Moreover, many programmes have seen recent reductions in uptake, and messaging is increasingly being employed to facilitate attendance.^38^, ^39^, ^40^ Our recommendations to utilise GP endorsement, which research has shown can increase attendance by 5%,^15^ and leverage behavioural science-informed messaging may help services counteract these trends.^41^ Furthermore, many of the recommendations look to reduce healthcare inequalities, such as requiring services and researchers to report inequality outcomes as standard. Whilst this has not been incorporated into existing mobile message guidance, it will help services address disparities in cohort identification for example at screening invitation, as well as ensuring services make adjustments for those with differing communication needs. This aligns with current national screening priorities to reduce these disparities.^42^^,^^43^

These principles are now being developed into national guidance, and therefore, will help stakeholders to effectively and appropriately implement messaging in screening, in a way that is consistent across different screening programmes. This is integral if services are to adhere to the recommendations by national screening reviews including implementing evidence-based initiatives to improve uptake and increase awareness of screening particularly amongst under-served groups.^7^ Moreover, our desirable item list helps set the agenda for future directions in screening messaging. This may include finding ways to make desired items, such as targeted messaging, more feasible to implement at scale, or focussing on using the increasing functionality of messaging services through app-integration or push notifications. Future work, however, will need to address whether these recommendations encompass all considerations as messaging technology continues to advance, and will also need to determine whether programme-specific extensions to our item list are required.

## Contributors

AA and NJW conceptualized the project. AA and GJ undertook data curation. AA, GJ, HA, VS, AS, MH and NJW all developed methodology and undertook project administration. AA and VS undertook formal analysis of the data, and this was validated by HA and GJ. AA undertook writing of the original draft, which was reviewed and edited by GJ, NJW and MH. AS and AD provided resources for this study. AD also provided supervision for the conduct of the study. AA, VS, HA, GJ and AD verified the underlying data. All authors read and approved the final version of the manuscript.

## Data sharing statement

De-identified data pertaining to this study and its outputs are available upon a reasonable request being made to the corresponding author and will be made available on the online registration service, Open Source Framework: https://osf.io/stf49/. Individual level data will not be shared as this poses a risk of de-identification given the limited number of experts enrolled.

## Declaration of interests

Professor Ara Darzi is Chair, and Hutan Ashrafian the Chief Scientific Officer of the Health Security initiative at Flagship Pioneering UK Ltd. Flagship Pioneering had no role in the development, conduct or analysis of the current study. No other authors declare conflicts of interest.
