# Supplementary material for: Investigating the national implementation of SMS and mobile messaging in population screening (The SIPS study)
Source: eBioMedicine. 2023 Jun 27;93:104685. doi: 10.1016/j.ebiom.2023.104685 (PMC10320235; doi:10.1016/j.ebiom.2023.104685)
Supplement: Supplementary material 1 [file mmc1.docx]

| **Author** | **Year** | **Type** | **Programme** | **Role of Message** | **Intervention** | **Author Conclusions** | **Areas for Consideration** |
| --- | --- | --- | --- | --- | --- | --- | --- |
| Hirst et al. | 2017 | RCT | Colorectal | Reminder to return kit | GP endorsed SMS at 7 weeks | Intervention did not lead to significant effect overall but increased uptake amongst first-time invitees. | Timing, Priming, Verification of numbers, Endorsement |
| Huf et al. | 2020 | RCT | Cervical | Reminder to book | Behavioural science informed messaging | Endorsed SMS reminder 3 weeks after invitation increased uptake by 18 weeks | Behavioural science, Endorsement, Verification of numbers, Preferences of communication |
| Huf et al. | 2017 | RCT | Breast | Reminder to attend | Two Behavioural science informed messages | Intervention did not lead to significant effect | Verification of numbers, Behavioural science, Endorsement |
| Icheku et al. | 2015 | Observational | Breast | Reminder to attend | SMS 1 week prior to appointment and further if DNA | Screening coverage increased by 2.54% with SMS | Verification of numbers, Effect on Inequalities, Privacy concerns, Evaluating acceptability |
| Kerrison et al. | 2015 | RCT | Breast | Reminder to attend, DNA SMS | SMS at 48h prior and further if DNA | Reminder increased attendance at first appointment offered. Effect size greatest amongst those from most deprived areas | Appointment details, Re-booking, Verification of numbers, Effect on inequalities |
| Ryan et al. | 2019 | Observational | Cervical | Reminder to book | App-based message with an ability to book | Over 10% of non-attenders booked an appointment | Effect on Inequalities, Signposting to non-app means of booking, Evaluating impact |

*48h- 48 hours, DNA- did not attend, RCT- randomised controlled trial, SMS- short message service*
