# Supplementary material for: Investigating the national implementation of SMS and mobile messaging in population screening (The SIPS study)
Source: eBioMedicine. 2023 Jun 27;93:104685. doi: 10.1016/j.ebiom.2023.104685 (PMC10320235; doi:10.1016/j.ebiom.2023.104685)
Supplement: Supplementary material 2 [file mmc2.docx]

| **Author** | **Year** | **Country** | **Type** | **Field** | **Role of Message** | **Areas for Consideration** |
| --- | --- | --- | --- | --- | --- | --- |
| CRISP | 2013 | USA | Guidance | Research | Mobile reminders, sharing information, health promotion | Evaluating impact, Maintaining security, Message tailoring, Timing, Consent and Opt-out, Effect on inequalities |
| Martinego et al. | 2020 | UK | Guidance | Primary Care | Mobile reminders, sharing information, health promotion | Preferences of communication, Maintaining security, Verification of numbers, Bi-directional messaging, Training needs, Privacy concerns, Audit of messaging processes, Integration with EHR, Messaging confirmation |
| MDU | 2021 | UK | Guidance | Primary Care | General communication | Preferences of communication, Consent and Opt-out, Maintaining security Privacy concerns, Evaluating acceptability, Communication needs |
| NHSX | 2022 | UK | Guidance | General | General communication | Maintaining security |
| RCN | 2016 | UK | Guidance | Nursing | Sharing information | Maintaining security, Evaluating impact, Accountability |
| Suggs et al. | 2015 | Qatar | Guidance | General | Health promotion, sharing information | Effect on Inequalities, New technologies, Evaluating impact, Evaluating acceptability, Communication needs, Behavioural science, Endorsement, Timing |
| US HHS | 2010 | USA | Guidance | General | Health promotion | Evaluating acceptability, Evaluating impact, Effect of inequalities, |
