# Supplementary material for: Investigating the national implementation of SMS and mobile messaging in population screening (The SIPS study)
Source: eBioMedicine. 2023 Jun 27;93:104685. doi: 10.1016/j.ebiom.2023.104685 (PMC10320235; doi:10.1016/j.ebiom.2023.104685)
Supplement: Supplementary material 3 [file mmc3.docx]

| **Domain** | **Item** | **Voting (%)** | | | **Designation** |
| --- | --- | --- | --- | --- | --- |
|  |  | R1  Imp. | R2  Imp. | R  Feas. |  |
| **Content** | Using concise simple language (reading age of 9) | 87⋅9 | - | 83⋅9 | 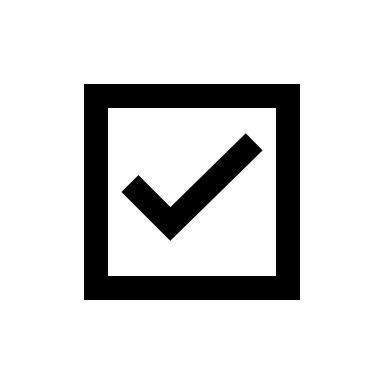 |
|  | Using non-technical language with factual, non-coercive information | 81⋅8 | - | 92⋅9 | 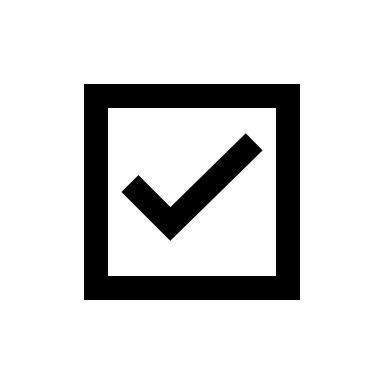 |
|  | Specifying the date, time (am/pm), location | 84⋅8 | - | 79⋅1 | 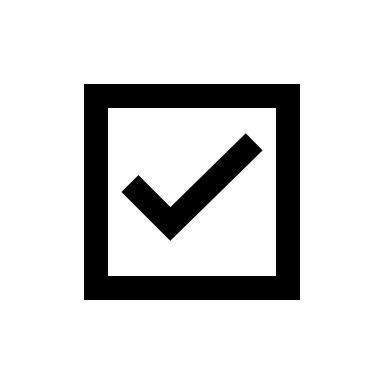 |
|  | Include additional information such as what to bring, or what to do, where possible. | 60⋅6 | 75⋅8 | 76⋅1 | 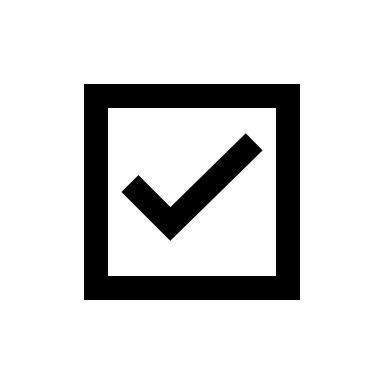 |
|  | Specifying who has sent the message (e.g. screening service or GP practice) and purpose | 90⋅9 | - | 92⋅9 | 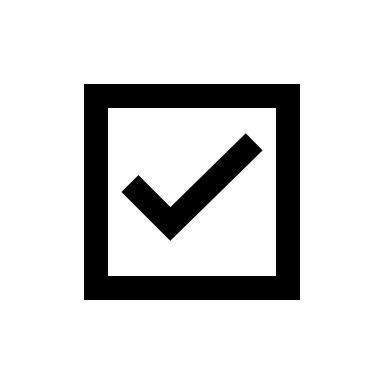 |
|  | Including weblinks to evidence or more information (e.g. screening website) | 84⋅8 | - | 88⋅9 | 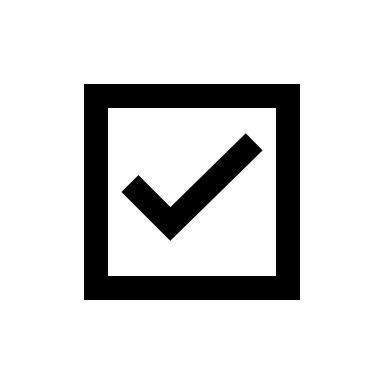 |
|  | Providing a telephone number to book | 81.8 | - | 81⋅8 | 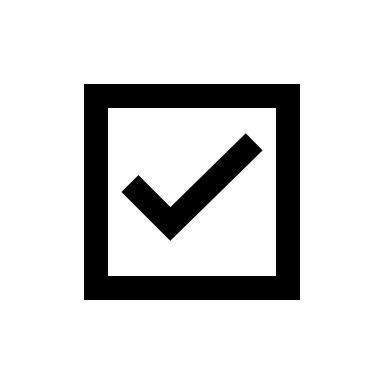 |
|  | Where appropriate using GP endorsement in reminder messages (e.g. [Practice name] encourages you to screen] | 72⋅7 | - | 81⋅3 | 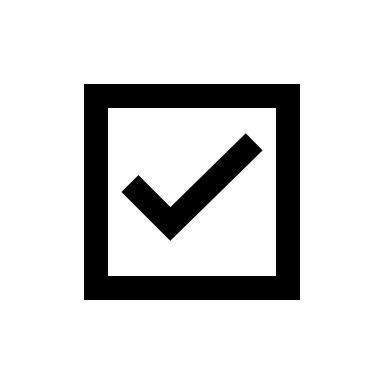 |
|  | Sending messages to facilitate attendance at screening (without being coercive), which could use behavioural science | 84⋅8 | - | 78.9 | 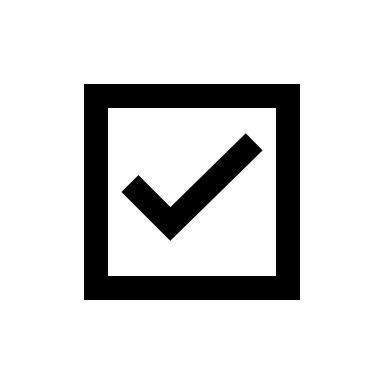 |
|  | Using Did Not Attend Messaging (DNA) messages for missed appointments | 60⋅6 | 84⋅8 | 75⋅0 | 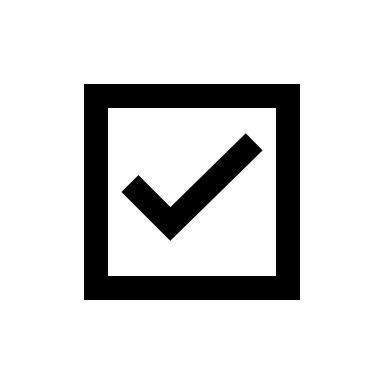 |
|  | Sending messages in English, but with language translations available (e.g. via weblink or by previous selection) | 87⋅9 | - | 63⋅6 | 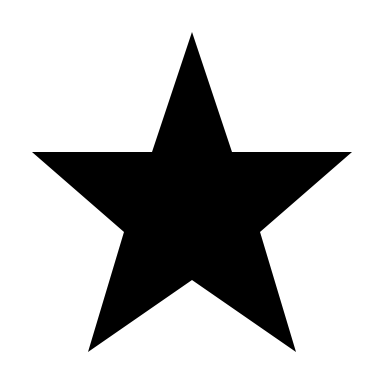 |
|  | Providing an ability to re-book in the message other than telephone no. (e.g. by text or weblink) | 72⋅7 | - | 39⋅4 | 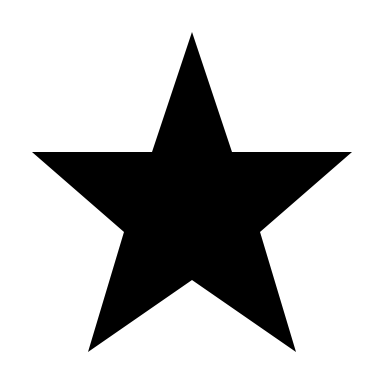 |
|  | Using messages tailored or targeted at certain groups (such as patients at higher risk of an illness) | 54⋅5 | 84⋅8 | 48⋅5 | 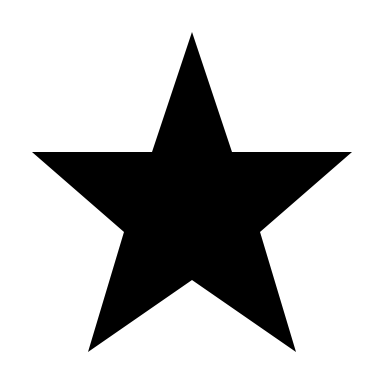 |
| **Timing** | 2 messages maximum should be sent at 1 time in the programme ideally | 57⋅6 | 78⋅8 | 78⋅8 | 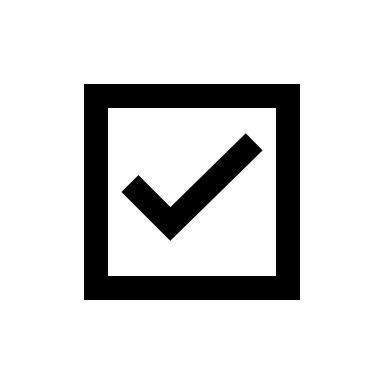 |
|  | Before an appointment 2 reminder messages should be sent at day 7 before then at day 2 before. | 51⋅5 | 78⋅8 | 78⋅8 | 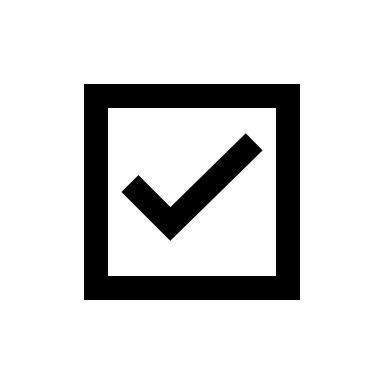 |
|  | Following an open invitation (e.g. to book an appointment) or sending of testing kit 3 messages should be sent if there has been no booking or returned kit. These will be on average 12 days, 20 days then 28 days after the invitation. | 54⋅5 | 72⋅7 | 72⋅7 | 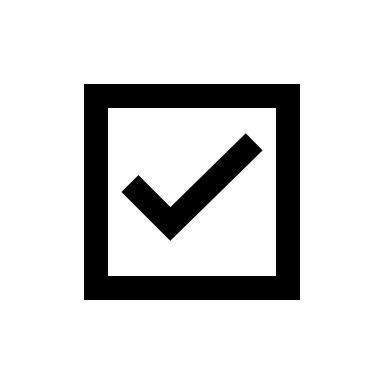 |
|  | Using confirmation texts immediately if a booking has been made or a kit has been received | 78⋅8 | - | 57⋅6 | 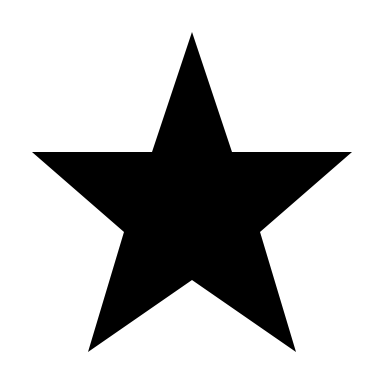 |
| **Delivery** | Flagging individuals who have who it might not be appropriate to message (e.g. following a miscarriage/patient passing away) | 81⋅8 | - | 46⋅9 | 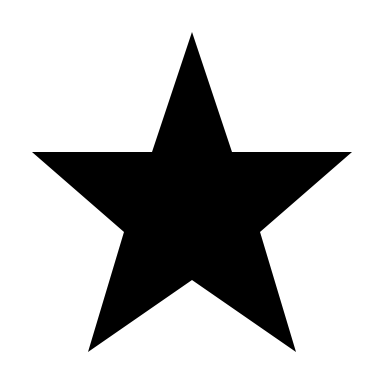 |
|  | Ensuring all services are integrated into the GP Spine to enable telephone number verification | 93⋅9 | - | 63⋅6 | 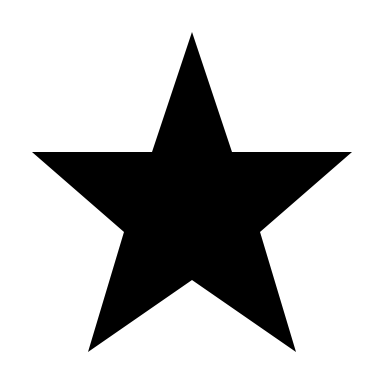 |
|  | Verifying numbers through direct contact with patients where possible | 81⋅8 | - | 50⋅0 | 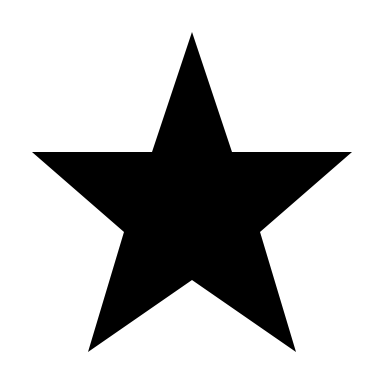 |
|  | Enabling limited bi-directional messaging service (e.g. for booking, confirming locations, organizing translations) | 48⋅5 | 72⋅7 | 36⋅4 | 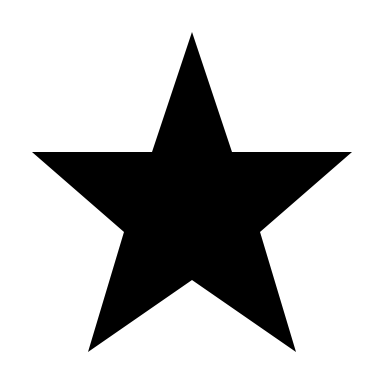 |
| **Evaluation** | Routinely evaluating the impact of new/different messages on regional healthcare inequalities | 90⋅9 | - | 75⋅0 | 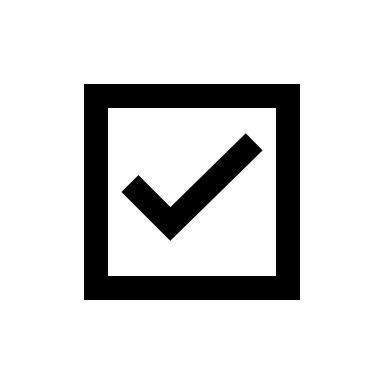 |
|  | Measuring user satisfaction by recording opt-out rates | 60⋅6 | 84⋅8 | 81⋅3 | 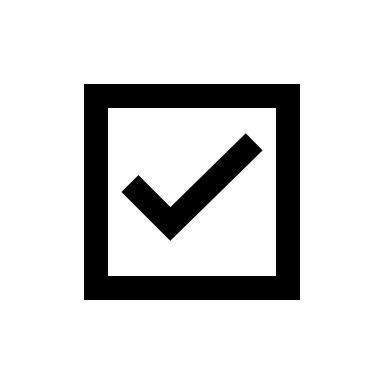 |
|  | If no existing pathway is available, periodically assessing usefulness of messages/satisfaction through other means (online, telephone and in writing) | 51⋅5 | 81⋅8 | 81⋅3 | 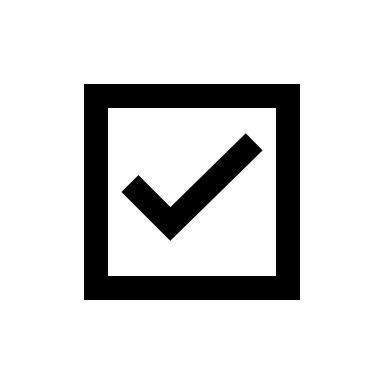 |
|  | To ensure ongoing acceptability of messages to the public, introducing ongoing testing (e.g. online A/B testing, or User-experience trials) | 63⋅6 | 78⋅8 | 72⋅7 | 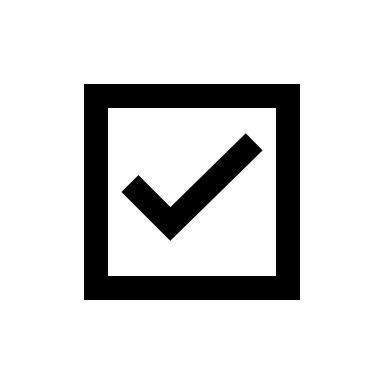 |
|  | Incorporating satisfaction measures into existing pathways (e.g. GP practices or NHSP Parent Survey) where possible | 75⋅8 | - | 42⋅4 | 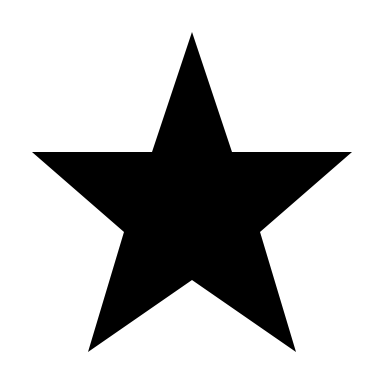 |
|  | Assessing measure mobile message delivery success reports and measure responses rates (e.g. in bi-directional messages, or appointment calls) | 84⋅8 | - | 62⋅5 | 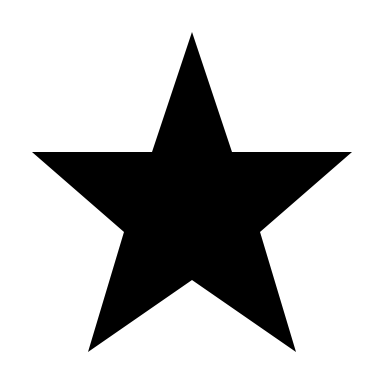 |
|  | When necessary using linked datasets (e.g. between screening services and GP data or hospital data) to facilitate the evaluation on healthcare inequalities | 90⋅9 | - | 51⋅5 | 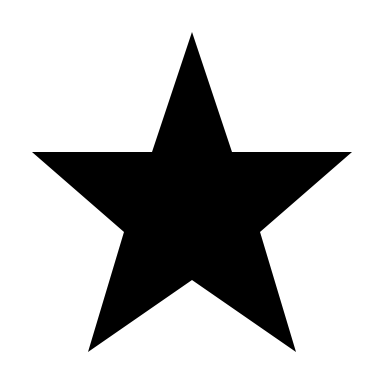 |
|  | Routinely collect measures of knowledge and attitudes (e.g. Decisional Conflict Scale) to screening to determine the effect on informed choice | 72⋅7 | - | 27⋅2 | 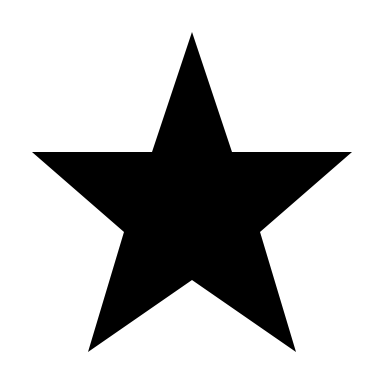 |
| **Security** | Maintaining consistency across media including publishing contact details/links on websites and in letters, so individuals can verify these as legitimate | 84⋅8 | - | 84⋅4 | 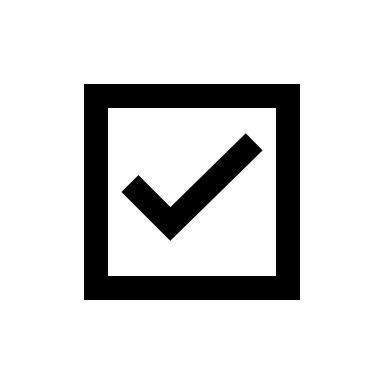 |
|  | Using MEF-registered (official) SenderIDs (e.g. “[Screeningservice] sent you a message”, as opposed to “[+4478…] sent you a message”) | 87⋅9 | - | 50⋅0 | 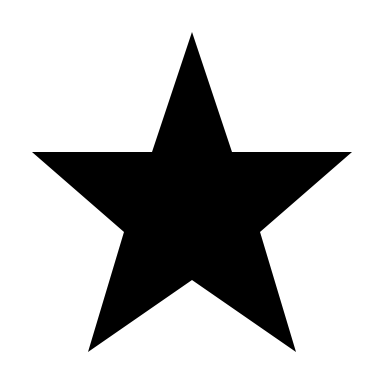 |
|  | Defining a wrong recipient message receipt as a reportable breach | 84⋅8 | - | 36⋅4 | 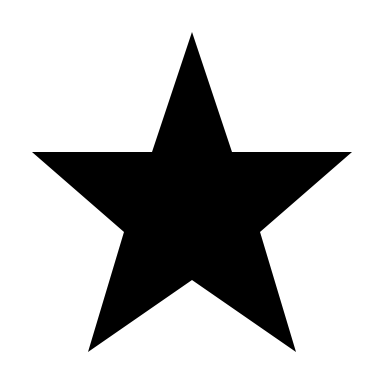 |
| **Research & Future** | Using experimental methods such as Randomised Controlled Trials to determine the impact of novel messages | 66⋅7 | 78⋅8 | 78⋅1 | 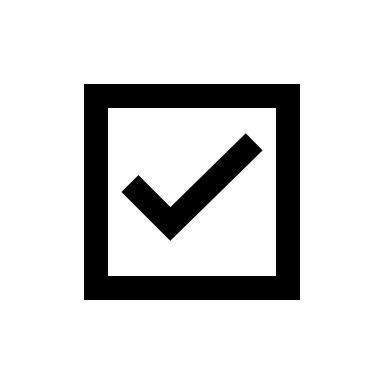 |
|  | Using online experimental methods such as A/B testing to determine the impact of novel message | 60⋅6 | 81⋅8 | 71⋅9 | 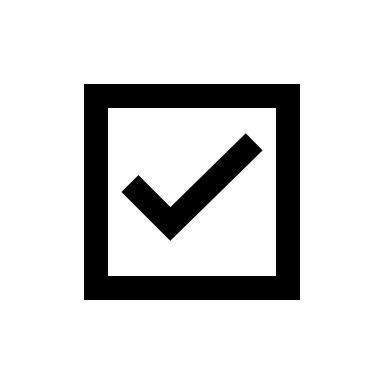 |
|  | Routinely report the outcomes of trials/research on population inequalities (e.g. between different demographics, and individuals with different health conditions) | 78⋅9 | - | 72⋅7 | 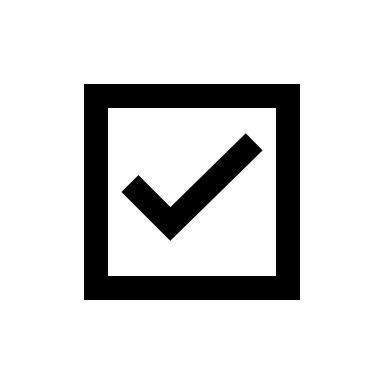 |
|  | Prior to large trials, new messages should ensure Patient and Public Involvement and qualitative measures are undertaken | 93⋅9 | - | 87⋅5 | 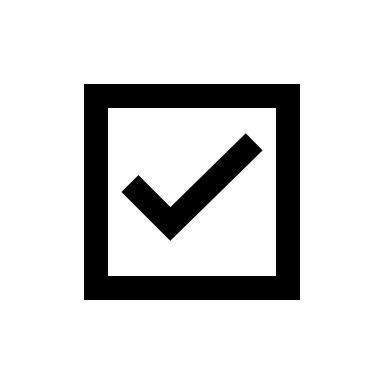 |
|  | Screening services/NHSE Publishing their research priorities, to enable researchers to focus upon relevant areas (this includes non-content related areas) | 84⋅8 | - | 79⋅1 | 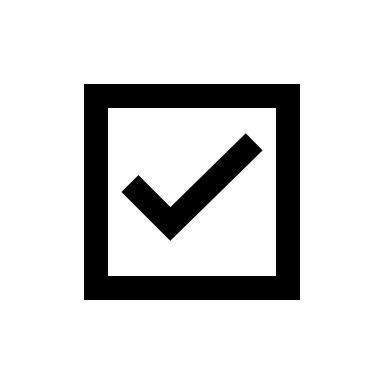 |
|  | Involving top-down infrastructure and governance support to facilitate research, including enabling trials across services/regions e.g. providing roadmaps for trial conduct, dissemination findings to stakeholders | 75⋅8 | - | 60⋅6 | 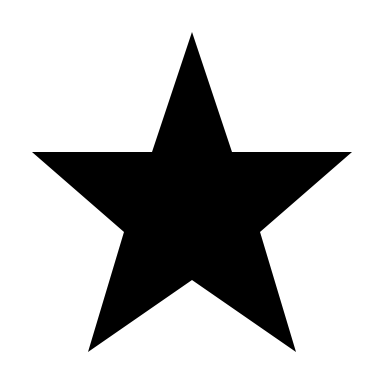 |
|  | Implementing fast-track processes to enable widespread testing for messages with trial evidence | 75⋅8 | - | 51⋅5 | 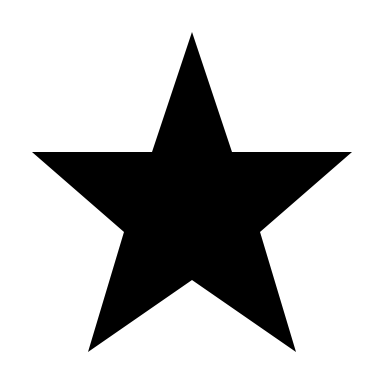 |
|  | Facilitate the examination of new technologies e.g NHS approved app-based integration or push notifications | 80⋅8 | - | 64⋅5 | 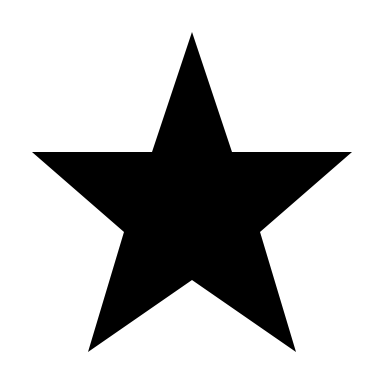 |


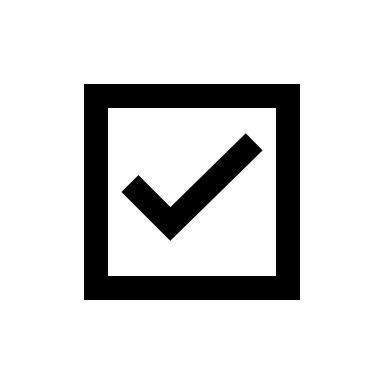

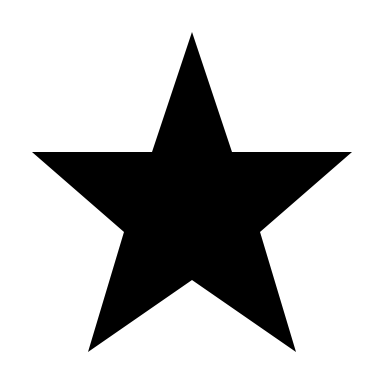
*DNA- did not attend, GP- general practitioner, NHSE- National Health Service England, NHSP- newborn hearing screening programme, R1 Imp- percentage of experts voting item as ‘important’ or ‘extremely important’ in round 1, R2 Imp- percentage of experts voting item as ‘important’ or ‘extremely important’ in round 2, R Feas.- percentage of experts voting item as ‘feasible’ or ‘absolutely feasible’ in the round that reached consensus with respect importance, core item, desirable item*
