# Supplementary material for: Investigating the national implementation of SMS and mobile messaging in population screening (The SIPS study)
Source: eBioMedicine. 2023 Jun 27;93:104685. doi: 10.1016/j.ebiom.2023.104685 (PMC10320235; doi:10.1016/j.ebiom.2023.104685)
Supplement: Supplementary material 4 [file mmc4.docx]

**Initial Item List**

In each case please your experience from the programmes you are familiar with.

Importance is defined as an item that is fundamental the effective use, governance or development of messages to the screening service

Feasibili**ty** is defined as an item that can be easily put into the current system without significant cost, time or logistical difficulty.

Below certain items there will be guides from a large patient group who were convened to give their opinions or experts such as the National Cybersecuity Services (NCSC). Note some items may contradict others, this to see if (1) the converse of an item is an important consideration and (2) as a sense check.

**Content of Messages**

1. Using concise simple language (reading age of nine)

2. Using non-technical language with factual, non-coercive information

3. Using messages two segments long (approx. 320 characters)

4. Enable messages to spread over multiple texts.

4. Sending messages in English, but with language translations available (e.g. via weblink or by previous selection)

5. Specifying the date, time (24h), location and what to bring

6. Specifying who has sent the message (e.g. screening service or GP practice), and purpose

7. Give a detailed purpose of the message

8. Using patient name in reminders

9. Including weblinks to evidence or more information (e.g. screening website)

10. Providing a telephone no. to re-book

11. Providing service addresses in reminder

12. Providing an ability to re-book in the message other than telephone no. (e.g by text or weblink)

13. Integrating message appointments into the MyGP app

14. Including one step opt-out (e.g via a link or text STOP to the number)

15. Where appropriate using GP endorsement in reminder messages (e.g [Practice name] encourages you to screen]

16. Using GP endorsement involving the GP/nurse name in preference to Practice name

17. Using generic endorsement to screen.

18. Using factual national cost information (e.g. missed appointments cost the NHS £X per year) in reminder messages

19. Using service specific/regional cost information (e.g. missed appointments cost your hospital £X per year).

20. Using opportunity cost information (e.g. missing appointments may mean delays in diagnosis) in reminder messages

21. Using limited Did Not Attend Messaging (DNA) messages

22. DNA messages stating the purpose and importance of the scan

23. DNA messaging to encourage to contact service/GP

24. Sending health promotion/improvement messages periodically regarding importance of screening, which could use behavioural science

25. Using messages tailored or targeted at certain groups

26. Enable individuals to determine the style of messaging they receive

27. Use of explicit statement invoking normative behaviour or social norms.

28. To ensure ongoing acceptability of messages to the public, introducing ongoing testing (e.g. online A/B testing, or User-experience trials)

29. Keep a record of the content of messages sent out previously

**Timing of Messages**

30. Sending messages during the working week (Mon-Fri 0900 to 1700)

31. Sending messages during weekends or out-of-hours

32. Send messages at a consistent time of day

33. Allowing preferences of time to be selected (either via website or text reply)


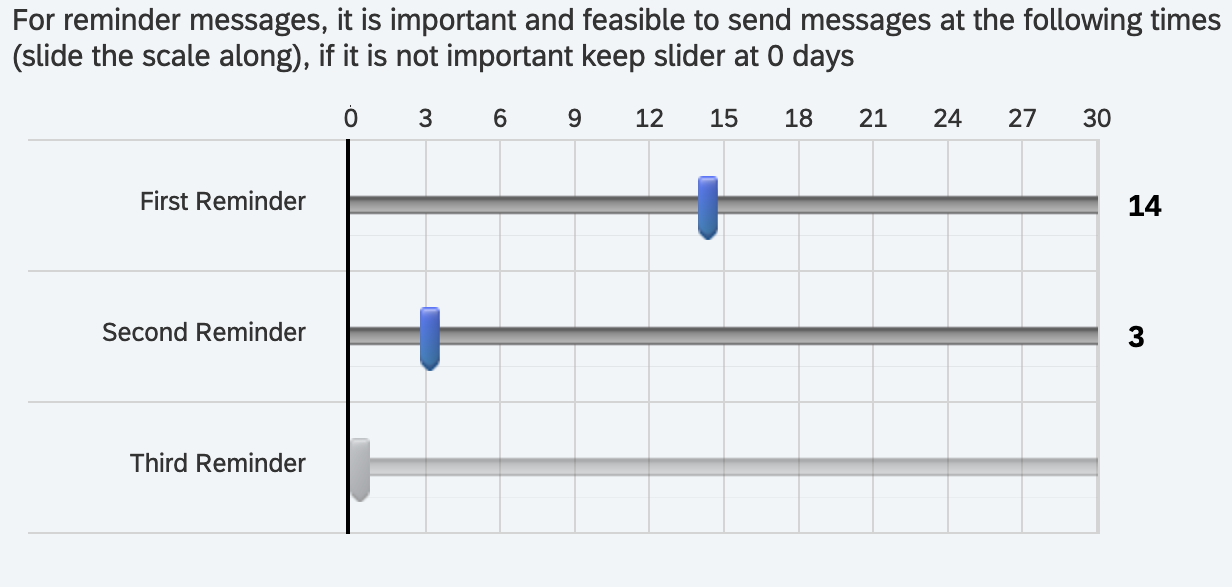
34-36. For reminder messages, it is important and feasible to send messages at the following times (slide the scale along), if is not important keep the slider at 0 days.


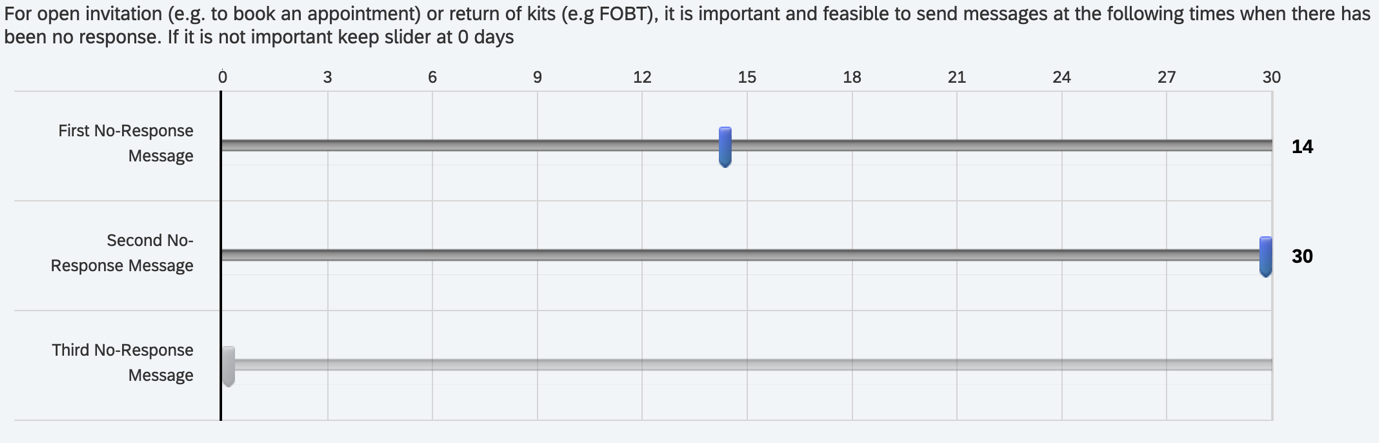
37-39. For open invitations (e.g. to book an appointment) or return of kits (e.g. FOBT), it is important and feasible to send messages at the following times when there has been no response. If it is not important keep the slider at 0 days.

40. Using confirmation texts immediately if a booking has been made or a kit has been received

41. Using confirmation texts immediately if an appointment has been attended.

42. Sending messages with status updates regarding results (e.g. results are expected in 2 weeks)

43. Record the timing of messages to facilitate future investigation into optimal schedules.

44. Coordinate timing of messages with those sent for other appointments to avoid happening at the same time

**Delivery of Messages**

45. Avoiding verifying the recipient identity through messages to avoid safeguarding or security issues

46. Enable recipient identity verification through messages

47. Flagging individuals who have opted out or who it might not be appropriate to message (e.g. following a miscarriage).

48. Flag individuals who have not attended previously

49. Ensuring all services are integrated into the GP Spine to enable telephone number verification

50. Enable linking to hospital services to enable telephone number verification

51. Verifying numbers through direct contact with patients where possible

52. Enabling voluntary online verification through screening websites/apps

53. Enable verification through response SMS

54. Enabling limited bi-directional messaging service (e.g. for functions such as booking, confirming locations, organizing translated messages)

55. Enable message-and-response services to be used for screening services

56. Using MEF-registered (official) SenderIDs (e.g [Screeningservice] sent you a message, as oppose [+4478…] sent you a message)

57. If an undeliverable message is received, flag this for verification

58. If a message requiring response (e.g. confirmation) is not answered, re-send messages requesting response.

**Security**

59. Reducing weblink/URL use- and where possible all should be human readable and easy to remember

60. Avoid including specific appointment details

61. Avoiding including contact details of services, which are better listed from official sources

62. Using bland messaging to avoid risk of breach (no results, patient addresses or personalised information)

63. Maintaining consistency across media including publishing contact details/links on websites and in letters, so individuals can verify these as legitimate

64. Not allowing messages to be received if IMSI (a phone’s ID) is roaming

65. Flag to the service if IMSI (a phone’s ID) is roaming

66. Defining a wrong recipient message receipt as a reportable breach

67. Log unsent/undeliverable messages

68. Avoiding shortened URL use in reminders

69. Use of links to non-screening service resources e.g. charities

70. Use of links to screening service resources hosted on 3^rd^ party sites

71. Adding security disclaimers/privacy wording into reminders

**Evaluation**

72. Measuring user satisfaction by Customer Effort (e.g. on a scale of ‘very easy’ to ‘very difficult’, how easy was it to interact with the screening message)

73. Use Net Promoter Scores (e.g. On a scale of 0 to 10, how likely is it that you would recommend this system to a friend or colleague)

74. Measure satisfaction by response to text

75. Measure satisfaction with system-reliability scales.

76. Measuring user satisfaction by opt-out number

77. Measure time to book, where applicable, as a determinant of message effectiveness

78. Use uptake rates as a measure of message effectiveness

79. Incorporating satisfaction measures into existing pathways (e.g. GP practices or NHSP Parent Survey) where possible

80. If no existing pathway is available, periodically assessing usefulness of messages/satisfaction through multiple means (online, telephone and in writing)

81. Use digital one click surveys or links to questionnaires

82. Assessing service outcomes measure SMS delivery success reports and measure responses rates (e.g. in bi-directional messages, or appointment calls)

83. Linking screening datasets to aid evaluation of national impact of messages and effect on healthcare inequalities

84. Examining healthcare inequalities directly through surveys and qualitative tools

**Future and Research Considerations**

85. Using experimental methods such as Randomised Trials to determine national message or novel messages

86. Use online experiments (including A/B testing) to determine national message or novel messages

87. Routinely report the outcomes of trials/research on population inequalities

88. As part of the design of new messages, evidencing Patient and Public Involvement and qualitative measures prior to trialing

89. Mandate that feasibility trials are undertaken prior to trials within screening services

90. Mandate that message-based trials are registered with an appropriate registry

91. Screening services are to be involved directly with research governance procedures

92. Publishing research priorities by screening services/PHE to enable researchers to focus upon relevant areas (this includes non-content related areas)

93. Involving top-down infra-structural and governance support to facilitate research, including enabling trials across services/regions

94. Screening services to facilitate the sharing of outputs from research with service users

95. Screening services to facilitate the sharing of outputs from research with other services

96. Implementing fast-track processes to enable real-world testing for messages with trial evidence

97. Facilitate collaborative funding applications with services for message-based research

98. Examining the use of new technologies such as message app-based integration

99. Examine the use of push notifications

100. Examine the use of bot technology or chat-bot responses

101. Examine use of calendar integration
